# Effects of acetazolamide on intracranial pressure and brain tissue oxygenation on patients with acute brain injury: A pilot physiological study

**DOI:** 10.14814/phy2.70159

**Published:** 2025-01-07

**Authors:** Anas Hachlouf, Claudia Stella, Irene Cavalli, Elisa Gouvêa Bogossian, Sophie Schuind, Marco Anderloni, Fabio Silvio Taccone

**Affiliations:** ^1^ Department of Intensive Care, Hôpital Universitaire de Bruxelles (HUB) Université Libre de Bruxelles (ULB) Bruxelles Belgium; ^2^ Department of Neurosurgery, Hôpital Universitaire de Bruxelles (HUB) Université Libre de Bruxelles (ULB) Bruxelles Belgium

**Keywords:** acetazolamide, acute brain injury, cerebral blood flow, cerebral oxygenation, intracranial pressure

## Abstract

The effect of acetazolamide on regional brain tissue oxygenation in patients with acute brain injury (ABI) is unknown. We studied adult patients with ABI who received acetazolamide as per the treating physician's decision and had ICP and brain oxygen pressure (PbtO_2_) monitoring. Baseline measurements of ICP, cerebral perfusion pressure (CPP), and PbtO_2_ were taken before administering acetazolamide; subsequent measurements were recorded every 5 min for a total of 20 min. Mean cerebral blood velocities (FVm) and pulsatility index (PI) were measured using transcranial color‐coded duplex (TCCD) sonography at baseline and after 20 min. Fourteen patients with subarachnoid hemorrhage (*n* = 6), traumatic brain injury (*n* = 7), and intracranial hemorrhage (*n* = 1) were included. Following administration of acetazolamide, ICP showed a significant increase within 20 min (*p* < 0.001), with no significant change in CPP (*p* = 0.08). PbtO_2_ demonstrated a significant increase (*p* < 0.001), with a noticeable change observed at 10 min after acetazolamide administration (15 [14–17] vs. 28 [26–30] mmHg). Additionally, FVm exhibited a significant increase (*p* < 0.001), and PI showed a reduction (*p* < 0.001). Administration of acetazolamide in ABI patients resulted in a significant increase in brain oxygenation, associated with a rise in ICP and FVm, suggesting increased cerebral volume and vasodilation.

## Graphical abstract

Administration of acetazolamide in patients with acute brain injury resulted in a significant increase in brain oxygenation, accompanied by a concurrent rise in ICP and flow velocities, suggesting increased cerebral volume due to vasodilation.

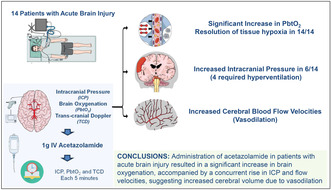

## 1 INTRODUCTION

Acute brain injury (ABI) encompasses a wide range of conditions that lead to brain damage, including traumatic brain injury (TBI), stroke, and hypoxic–ischemic encephalopathy. Following the primary insult, the brain enters a vulnerable state in which secondary brain injury can significantly worsen the initial damage and contribute to long‐term neurological deficits (Lazaridis et al., 2019). Among the various forms of secondary brain injury, intracranial hypertension and tissue hypoxia are common complications in severe ABI cases, both of which are associated with impaired cerebral perfusion, exacerbated ischemia, and diffuse neuronal damage (Oddo et al., 2011).

Effective management of these complications is critical for improving patient outcomes in ABI. Advanced neuromonitoring techniques have become essential in the care of critically ill ABI patients, enabling continuous, real‐time assessment of intracranial pressure (ICP) and brain oxygenation (PbtO_2_) (Oddo & Bösel, 2014). Early detection of elevated ICP and low PbtO_2_ values can facilitate the implementation of timely, protocolized interventions tailored to the individual patient's needs (Chesnut et al., 2020; Hawryluk et al., 2019). In particular, interventions aimed at improving PbtO_2_ generally focus on optimizing oxygen delivery (e.g., increasing oxygen administration, enhancing blood pressure, or administering red blood cell transfusions) or reducing oxygen consumption (e.g., sedation, neuromuscular paralysis, or hypothermia) (Chesnut et al., 2020; Godoy et al., 2023). However, the optimal selection of therapy to rapidly improve PbtO_2_ remains a topic of ongoing debate.

Alkalemia is not uncommon in brain‐injured patients, often due to compensatory mechanisms such as prolonged mild hyperventilation, the use of diuretics, or hypovolemia (Esnault et al., 2019). One of the key ways in which alkalemia affects cerebral oxygen delivery is by altering the oxygen‐hemoglobin dissociation curve. Specifically, alkalemia shifts the curve to the left, increasing hemoglobin's affinity for oxygen, which impairs oxygen unloading at the tissue level (Warner et al., 1987). Additionally, alkalemia may lead to cerebral vasoconstriction, which reduces cerebral blood flow (CBF) and further exacerbates tissue hypoxia (Hamilton et al., 2004).

Acetazolamide is the predominant inhibitor of carbonic anhydrase and is extensively utilized for metabolic alkalosis (Van Berkel & Elefritz, 2018). In the central nervous system, carbonic anhydrase is expressed in various cell types including neurons, oligodendrocytes, astrocytes, and choroid plexus cells; as such, acetazolamide can influence brain hemodynamics through various mechanisms, particularly its role in modulating pH homeostasis (Shukralla et al., 2022). By acidifying the extracellular and possibly intracellular environment (Heuser et al., 1975; Severinghaus & Cotev, 1968), acetazolamide induces arteriolar vasodilation, leading to an increase in CBF. Although some studies have assessed the cerebral vasomotor reactivity (VMR) after acetazolamide administration in patients with ABI (e.g., the ability of cerebral blood vessels to adjust their tone in response to changes in brain tissue pH) (Kitahara et al., 1996; Mokri, 2001; Szabo et al., 1997; Tanaka et al., 1998; Wolf, 2015), no studies have specifically investigated the drug effects on ICP.

Few studies have investigated the effects of acetazolamide on brain oxygenation. Some studies have demonstrated an increase in CBF and jugular venous oxygen saturation (SvjO_2_), while maintaining a constant cerebral metabolic rate for oxygen (CMRO_2_) in patients without brain injury (Posner & Plum, 1960; Vorstrup et al., 1984). These findings were further supported by another study (Kaminogo et al., 1995), which assessed the changes in CBF and regional oxygen saturation (rSO_2_), measured with near‐infrared spectroscopy, following acetazolamide administration in patients with acute ischemic stroke and healthy volunteers. In particular, a significant elevation in rSO_2_, which was well correlated with the increase in CBF, was observed. However, both SvjO_2_ and rSO_2_ have important limitations, including the absence of defined thresholds for tissue hypoxia, the potential contamination of measurements from extra‐cerebral blood, and the lack of specifically targeting at‐risk areas of the brain (Oddo & Bösel, 2014). These artifacts and limitations can lead to erroneous interpretation of the available findings. Therefore, it is important to utilize complementary tools and techniques to obtain a more comprehensive assessment of cerebral oxygenation, such as brain oxygen pressure (PbtO_2_) monitoring (Oddo & Bösel, 2014).

The aim of this study was therefore to assess the effects of acetazolamide on ICP and PbtO_2_ in ABI patients. We hypothesized that acetazolamide would exert a vasodilatory effect, thereby increasing both ICP and PbtO_₂_; however, the extent of these effects is unknown and needs to be precisely quantified.

## 2 METHODS AND MATERIALS

### 2.1 Study design

This is a single‐center analysis of prospectively collected data conducted in the ICU of the Erasme University Hospital in Brussels (Belgium) from January 2017 to March 2023. The study protocol was approved by the local ethics committees (Comité d'Ethique de l'Hôpital Erasme – study ID: SRB2022383); a written delayed consent was obtained from next of kin, as the administration of acetazolamide was determined independently of the study, and the repeated and serial assessments of clinically relevant variables, including transcranial Doppler and gas analyses, were part of a standardized data collection process for various interventions administered to patients undergoing invasive multimodal neuromonitoring in the ICU.

Patients meeting the following eligibility criteria were included in the study: (a) age >18 years; (b) admission to the intensive care unit (ICU) for ABI (including SAH, ICH, and TBI); (c) requirement for mechanical ventilation in controlled mode (i.e., without spontaneous breathing); (d) continuous monitoring of ICP and PbtO_2_; (e) baseline ICP below 20 mmHg; (f) administration of acetazolamide (Diamox®, Mercury Company Ltd., London, UK) as per the treating physician's decision (i.e., for the treatment of metabolic alkalosis). Patients admitted for other forms of ABI or on spontaneous breathing and individuals with unreliable PbtO_2_ or ICP readings were excluded from the study.

### 2.2 Measurements and data collection

Demographic information, including age and sex, as well as the type of ABI, were collected for all patients. The initial severity of the disease was assessed using the Glasgow Coma Scale (GCS) and radiological findings, such as the Marshall classification for TBI patients and the modified Fisher scores for SAH patients upon admission. Additionally, important outcomes including the length of stay in the intensive care unit (ICU), in‐hospital mortality, and the Glasgow Outcome Scale (GOS) at the time of hospital discharge were reported.

The ongoing treatments at enrollment, including mechanical ventilation, vasoactive drugs, sedation, and analgesia, were collected. Baseline clinical and laboratory findings, such as temperature and plasma concentrations of hemoglobin, sodium, and glucose, were also documented. Continuous measurement of PbtO_2_ was performed using an intraparenchymal probe (Integra LifeSciences Corporation, Plainsboro, NJ, USA), which was carefully positioned near the site of brain injury following the local protocol. Intracranial pressure was also continuously monitored using either an intraparenchymal or intraventricular probe. Cerebral perfusion pressure (CPP) was calculated as the difference between the mean arterial pressure (MAP) and ICP (CPP = MAP – ICP). Measurements of PbtO_2_, ICP, and CPP were recorded at baseline (i.e., before acetazolamide administration) and every 5 min for a duration of 20 min following an intravenous bolus of 1 g acetazolamide; values were averaged over a period of 10 s, as they were recorded and stored in the patient data monitoring system. The PbtO_2_/PaO_2_ ratio was calculated at baseline and at 20 min after acetazolamide administration.

Transcranial color‐coded duplex (TCCD) sonography was utilized to assess cerebral flow velocities in the middle cerebral artery (MCA), including systolic flow velocity (FVs), diastolic flow velocity (FVd), and mean flow velocity (FVm), using a 2‐MHz transducer. The target MCA for initial assessment was chosen based on the side ipsilateral to the injury or aneurysm location, as this provides essential information on CBF in the affected hemisphere. In cases where the injury involved both hemispheres or there was no clear lateralization, TCCD was performed bilaterally, with the MCA demonstrating the lowest FVm selected for further monitoring. The FVm [(FVs + FVd *2)/3] and the pulsatility index (PI) [(SFV‐DFV)/MFV] were automatically calculated by the echography software (Philips iE33, Paris, France). In particular, changes in FVm were used as a surrogate for CBF, based on the assumption that the cross‐sectional area of the MCA remained stable (e.g., an increase in CBF would be reflected by an increase in FVm). Additionally, the PI was used as an indicator of cerebrovascular resistance (e.g., arteriolar vasoconstriction would correspond to an increase in PI). The transtemporal acoustic window was used for insonation of the MCA; cerebral ultrasound imaging of the brain parenchyma, along with identification of characteristic flow patterns and Doppler signals, guided selection of an MCA segment approximately 1 cm distal to the internal carotid artery bifurcation. Measurements were taken at each time point, over the same 10‐s period than other variables, as they were recorded and stored in the patient data monitoring system without a dedicated headset for probe stabilization; however, the angle of insonation was consistently maintained below 15° to minimize its effect on absolute FVm values. Operators adjusted the probe position until the strongest and most stable signal was obtained.

TCCD measurements were obtained at baseline and 20 min after the administration of acetazolamide, together with arterial blood gas analyses and end‐tidal carbon dioxide (etCO_2_). The decision to conduct the study over a 20‐min period was made to minimize the likelihood of other interventions becoming necessary during the observation, which could potentially confound the measured variables. Using arterial blood gas analyses, the p50, for example, the oxygen tension when hemoglobin is 50% saturated with oxygen, was calculated using the Hill equation modified by Dash (Dash et al., 2016). The presence of brain tissue hypoxia was defined as PbtO_2_ <20 mmHg for at least 5 min; intracranial hypertension was defined as an ICP >20 mmHg for at least 5 min.

### 2.3 Study outcomes

The primary outcome of this study was to evaluate the changes in PbtO_2_ and ICP following the administration of acetazolamide. The secondary outcomes included investigating the correlation between changes in PbtO_2_ and FVm, as well as examining the variations in biochemical variables, such as pH, PaCO_2_, PaO_2_, lactate, and p50. Also, the comparison of PbtO_2_ values over time between traumatic and non‐traumatic injury was assessed.

### 2.4 Statistical analysis

All statistical analyses were conducted using GraphPad Prism software (version 2023, GraphPad Software). Descriptive statistics were calculated for all variables. All variables were reported as median and interquartile range (IQR). Differences between paired measures were assessed using Wilcoxon's test. Repeated measures were compared using non‐parametric Friedman's test, with Dunn's multiple comparison test post hoc analysis. Relative changes in PbtO_2_ [(PbtO_2_ at 20 min – PbtO_2_ at baseline)/PbtO_2_ at baseline * 100, %], in ICP [(ICP at 20 min – ICP at baseline)/ICP at baseline * 100, %], and in FVm [(FVm at 20 min – FVm at baseline)/FVm at baseline * 100, %] were also computed; linear correlations were determined using Pearson's correlation coefficient. Statistical significance was defined as a *p* value of less than 0.05.

## 3 RESULTS

### 3.1 Characteristics of the study participants

During the study period, a total of 143 patients underwent monitoring of ICP and PbtO_2_; among them, 19 patients received acetazolamide; however, only 14 patients met the eligibility criteria, as the drug was administered while invasive monitoring was still available. The characteristics of the study population are presented in Table 1. The majority of the patients were male, with a median age of 44 [35–51] years. Half of the patients had traumatic brain injury (TBI) (*n* = 7, 50%). The Glasgow Coma Scale (GCS) score upon admission was 7 [5–8], and the median time from ICU admission to acetazolamide administration was 7 [6–8] days. The length of stay in the ICU was 21 [17–28] days, the Glasgow Outcome Scale (GOS) score at hospital discharge was 3 [1–4], and the in‐hospital mortality rate was 29%.

**TABLE 1:** Characteristics of the study population at baseline.

Variables	Total cohort (*n* = 14)
Demographics	
Age, years	44 [35–51]
Male gender, *n* (%)	9 (64%)
Acute brain injury	
Initial GCS	7 [5–8]
SAH, *n* (%)	6 (43%)
mFisher CT classification	4 [4–4]
TBI, *n* (%)	7 (50%)
Marshall CT classification	4 [3–5]
ICH, *n* (%)	1 (7%)
Temperature (°C)	37.1 [36.5–37.2]
Hemoglobin (g/dL)	9.1 [8.9–9.6]
Sodium (mEq/L)	139 [138–141]
Glucose (mg/dL)	123 [119–144]
Vasopressors, *n* (%)	14 (100%)
Inotropes, *n* (%)	0 (0%)
Sedatives, *n* (%)	14 (100%)
Propofol	14 (100%)
Midazolam	1 (7%)
Ketamine	4 (29%)
Opioids	13 (93%)
NMBA	14 (100%)
PEEP, cmH_2_O	8 [6–10]
Heart Rate, bpm	89 [78–97]

*Note*: Values are expressed as count (%) or median [interquartile range].

Abbreviations: CT, Computed Tomography; GCS, Glasgow Coma Scale; ICH, Intracranial Hemorrhage; ICU, Intensive Care Unit; NMBA, Neuro‐Muscular Blocking Agents; PEEP, Positive End‐Expiratory Pressure; SAH, Subarachnoid Hemorrhage; TBI, Traumatic Brain Injury.

### 3.2 Brain oxygenation and intracranial pressure

**FIGURE 1:**
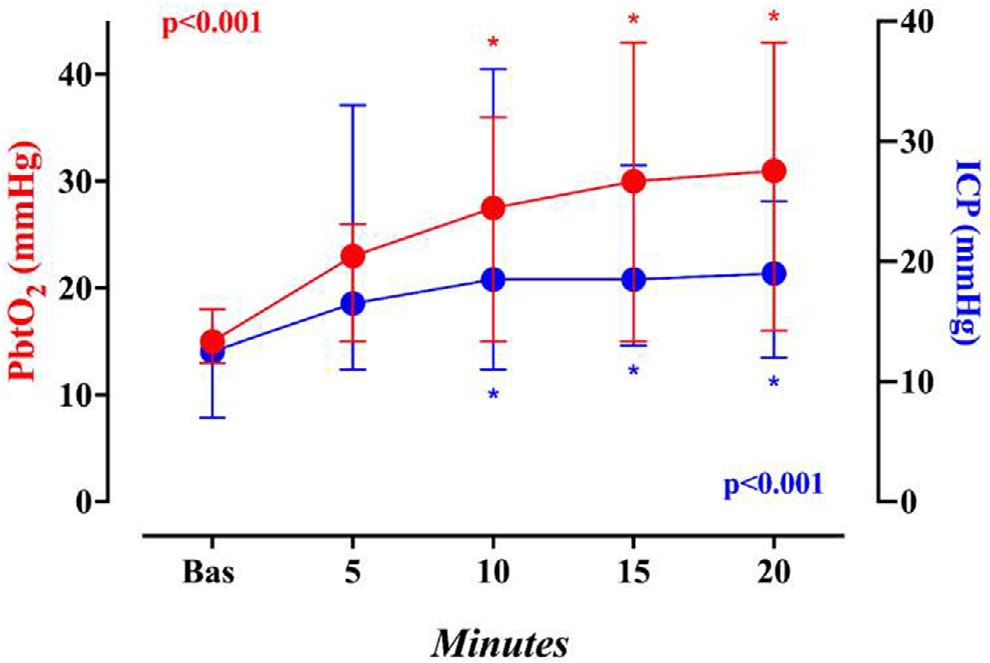
Changes in intracranial pressure (ICP) and brain oxygen pressure (PbtO2) at baseline and 5, 10, 15, and 20 min after acetazolamide administration. *p* values are derived from Friedman's test. * Indicates significant difference versus baseline (Dunn's test).

The baseline values for PbtO_2_ and ICP were 15 [14–17] mmHg and 13 [9–15] mmHg, respectively (Table 2 and Figure 1). Following administration of acetazolamide, there was a significant increase in PbtO_2_ over time (*p* < 0.001), which was already significant at 10 minutes (28 [26–30] mmHg) and remained stable thereafter (Figure 1). Only one patient did not show any change in PbtO_2_ after acetazolamide administration, while in the other thirteen PbtO_2_ exceeded 20 mmHg over time.

**TABLE 2:** Repeated measurements of different variables over time.

	Baseline	5 min	10 min	15 min	20 min	*p* Value
PbtO_2_, mmHg	15 [15–17]	23 [22–24]	28 [26–29]	30 [29–32]	31 [29–35]	<0.001
ICP, mmHg	13 [10–14]	17 [15–18]	19 [17–21]	19 [17–24]	19 [16–21]	<0.001
CPP, mmHg	85 [81–91]	85 [78–88]	84 [76–87]	83 [79–89]	83 [81–87]	0.08
SFV, cm/s	109 [98–125]	–	–	–	171 [157–183]	<0.001
DFV, cm/s	51.00 [49–58]	–	–	–	100 [92–107]	<0.001
MFV, cm/s	72 [67–78]	–	–	–	127 [116–130]	<0.001
PI	0.74 [0.69–0.89]	–	–	–	0.58 [0.51–0.69]	<0.001
pH	7.44 [7.43–7.45]	–	–	–	7.38 [7.37–7.41]	0.003
PaCO_2_, mmHg	50 [49–55]	–	–	–	51 [48–57]	0.32
PaO_2_, mmHg	110 [103–135]	–	–	–	119 [112–151]	0.02
Lactate, mmol/L	0.8 [0.7–0.9]	–	–	–	0.6 [0.5–0.7]	0.45
PbtO_2_/PaO_2_	0.14 [0.12–0.15]	–	–	–	0.26 [0.23–0.29]	<0.001
p50	25.5 [24.1–30.4]	–	–	–	26.8 [24.8–30.2]	0.002

*Note*: Data are expressed as median and interquartile ranges. Analyses are performed using Friedman's test or Wilcoxon's test, as appropriate. *p* values <0.05 are significant.

Abbreviations: FVd, diastolic Flow Velocity; FVm, mean Flow Velocity; FVs, Systolic Flow Velocity; ICP, intracranial pressure; p50, pressure of oxygen at which 50% of hemoglobin is saturated; PbtO_2_, brain oxygen pressure; PI, Pulsatility Index.

Similarly, ICP increased over time (*p* < 0.001 – Figure 1), with a significant rise observed after 10 min (19 [16–22] mmHg), which then remained stable. In eight patients, ICP remained below 20 mmHg over the observational period (maximum ICP value ranging from 13 to 19 mmHg); in six patients, the ICP exceeded this threshold, with maximum values ranging from 22 to 36 mmHg, most of which occurred within 10 min following acetazolamide administration. It is worth noting that four of these six patients required an increased respiratory rate (median of 3 [ranges: 2–3]) to bring ICP levels below 25 mmHg. At the end of the observational period, one patient had still an ICP of 25 mmHg, while other values ranged from 18 to 21 mmHg. No significant changes in cerebral perfusion pressure (CPP) were observed over time (*p* = 0.08).

### 3.3 Brain oxygenation and cerebral blood velocity

**FIGURE 2:**
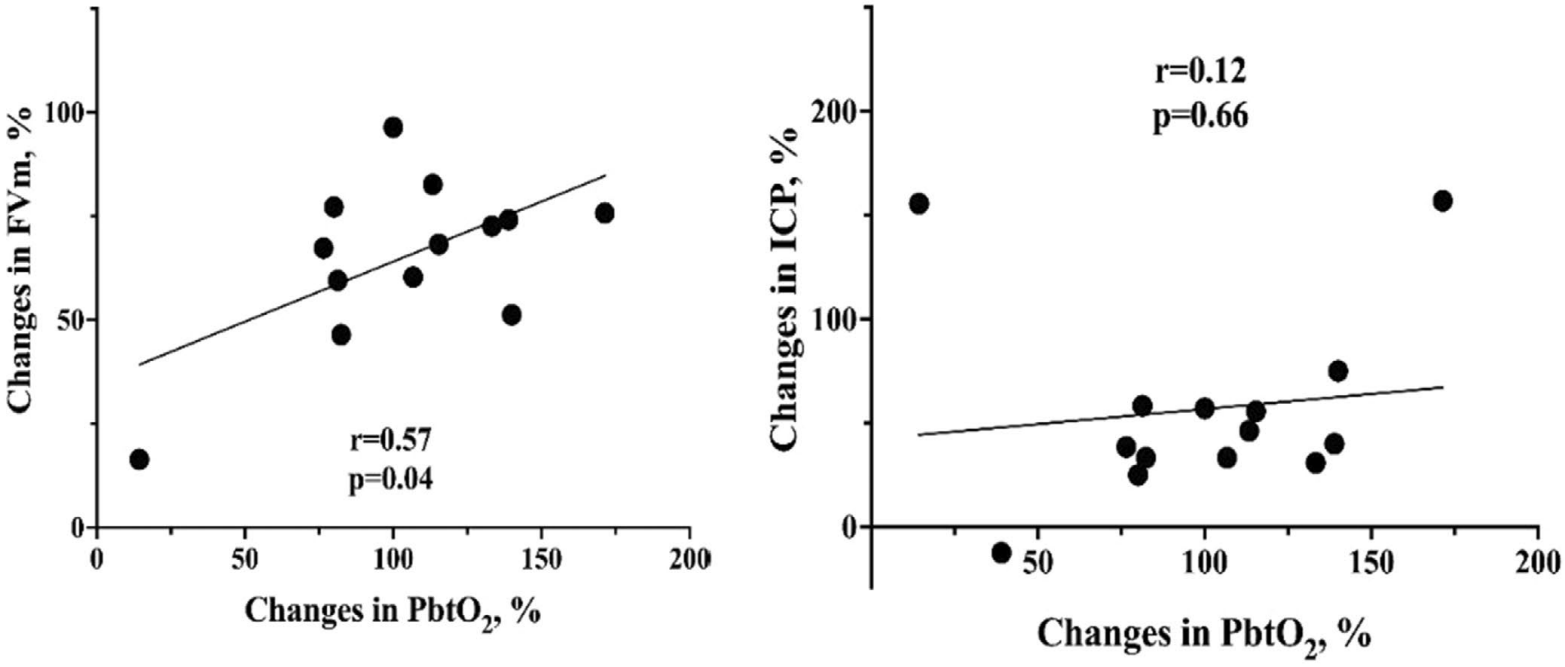
Pearson's correlation coefficient between changes in brain oxygen pressure (PbtO2), cerebral mean flow velocities (FVm) and intracranial pressure (ICP) 20 min after acetazolamide administration. *p* values <0.05 are significant.

Variations in FVm were measured in 13 patients, as one patient had no transtemporal window to perform TCCD; FVs, FVd, and FVm all increased after acetazolamide administration (Table 2). Also, the PI was significantly reduced. A significant correlation between changes in PbtO_2_ and FVm (*r* = 0.57, *p* = 0.04) was observed (Figure 2), while no correlation between changes in PbtO_2_ and ICP was found.

### 3.4 Other secondary outcomes

**FIGURE 3:**
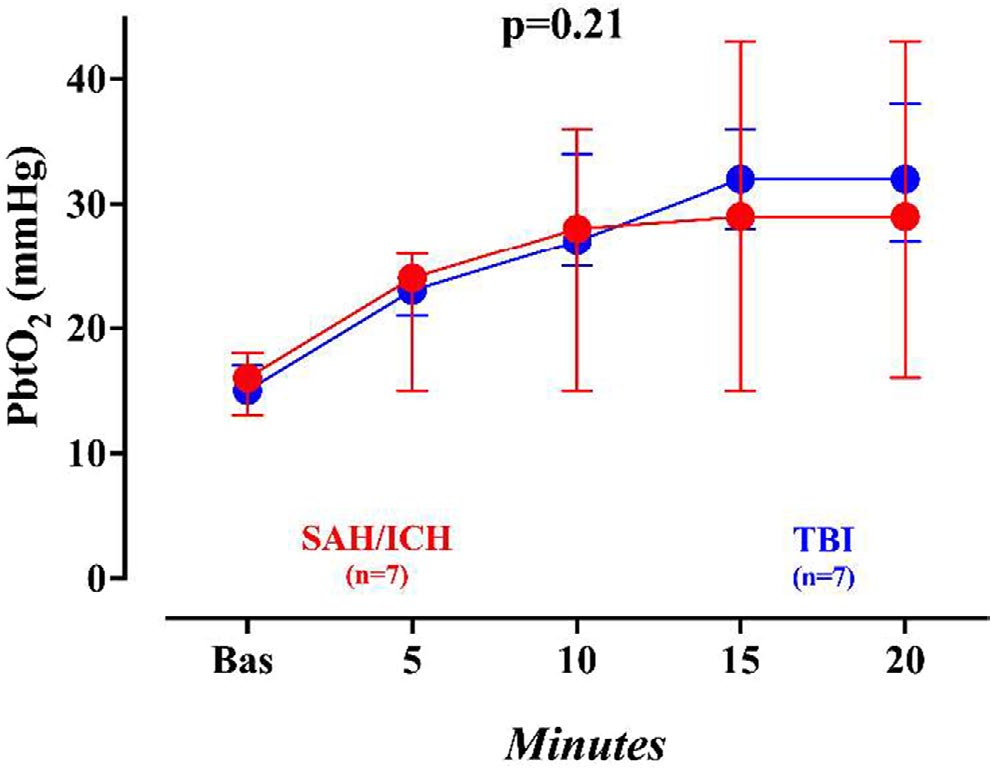
Changes in brain oxygen pressure (PbtO2) over time in traumatic (TBI) and non‐traumatic (SAH/ICH) brain injury. *p* Values are derived from Friedman's test. ICH, intracranial hemorrhage; SAH, subarachnoid hemorrhage; TBI, traumatic brain injury.

A significant increase in PaO_2_ and a decrease in pH were observed after acetazolamide administration; no significant changes in PaCO_2_ and lactate levels were observed; p50 also significantly increased after acetazolamide injection. No difference in PbtO_2_ values over time between patients with traumatic or non‐traumatic brain injury was observed (Figure 3).

## 4 DISCUSSION

This study suggested that acetazolamide can effectively increase brain oxygenation in patients with acute brain injury and tissue hypoxia. The mechanism behind this effect is primarily attributed to cerebral vasodilation, leading to an increase in cerebral blood flow (CBF) velocities, but a reduction in the hemoglobin‐oxygen affinity was also observed. Additionally, a notable increase in intracranial pressure (ICP) was observed, likely due to the expansion of intracerebral volume, specifically the arterial component. These findings highlight the potential of acetazolamide as a therapeutic intervention to improve brain oxygenation in selected ABI patients.

At the best of our knowledge, this is the first report on the effects of acetazolamide on PbtO_2_. Monitoring of PbtO_2_ plays a crucial role in the management of ABI patients by providing early detection of brain tissue hypoxia and tailoring treatment strategies aiming at minimizing secondary brain injury (Oddo & Bösel, 2014). In TBI patients, a protocol implementing PbtO_2_‐guided therapy resulted in the reduction of the incidence of brain hypoxia, when compared to ICP‐guided therapy alone (Okonkwo et al., 2017). Moreover, two meta‐analyses have further supported the benefits of combined ICP/PbtO_2_‐guided therapy in improving neurological outcomes compared to standard ICP‐guided therapy in TBI patients (Gouvêa Bogossian et al., 2022; Xie et al., 2017). In SAH patients, low PbtO_2_ levels have been associated with various pathological pathways, including reduced CBF, acute lung injury leading to hypoxemia, and anemia (Gouvea Bogossian et al., 2023). Addressing these factors and implementing strategies to increase brain perfusion can effectively elevate PbtO_2_ levels in certain SAH patients and potentially improve outcome, at least in more severe cases (Gouvea Bogossian et al., 2021). These findings underscore the importance of monitoring and incorporating PbtO_2_ measurements into clinical management strategies for individualized and targeted interventions to improve cerebral perfusion and mitigate the risk of brain hypoxia in this setting.

One potential approach to increasing PbtO_2_ levels is to induce vasodilation through the use of hypercapnia, which results in the dilation of cerebral arterioles and an increase in CBF (Westermaier et al., 2016). The mechanism behind CO_2_‐induced vasodilation involves changes in the pH of the cerebrospinal fluid (CSF) and the release of vasodilatory mediators such as nitric oxide (NO), adenosine, and prostaglandins (Harper & Glass, 1965). However, in an animal model, the effects of normoxic hypercapnia were observed to persist for a duration of 3 h; after this period, an adaptive response occurred, that is, CSF pH being initially acidotic subsequently increased over time, accompanied by a rise in the concentration of bicarbonate ions, leading to a decrease in CBF and an increase in cerebrovascular resistance (Warner et al., 1987). As such, this compensatory mechanism (i.e., bicarbonate‐buffering via the reversible reaction between carbonic acid and bicarbonate ions) would partially or totally restore the perivascular acid–base balance into the brain tissue, reducing the effects of hypercapnia on cerebral vasodilation and require even further increase in PaCO_2_ levels to improve brain perfusion. In this setting, acetazolamide acts as an inhibitor of carbonic anhydrase, which is involved into the reversible conversion of carbon dioxide (CO_2_) to bicarbonate (HCO_3_−); by inhibiting carbonic anhydrase, acetazolamide increases the concentration of H^+^ in the perivascular space, leading to cerebral vasodilation (Severinghaus & Cotev, 1968; Shukralla et al., 2022). Moreover, as carbon anhydrase is largely expressed in red blood cells, a systemic reduction in pH might lead to a rightward shift of the oxygen‐hemoglobin dissociation curve (i.e., reduced affinity of hemoglobin for oxygen, with enhanced oxygen unloading in peripheral tissues) (Hamilton et al., 2004). Our study findings support the notion that acetazolamide administration resulted in cerebral vasodilation, as evidenced by increased FVm and decreased PI, leading to an increase in intracerebral volume and CBF. These effects had two distinct consequences; the first one is the ICP increased; as a result, minute ventilation had to be adjusted in four patients to mitigate this phenomenon. This is in contrast with several studies showing a reduction in ICP after acetazolamide injection, by targeting the choroid plexus and decreasing CSF secretion and/or aquaporins channels inhibition (Chaaban et al., 2013; Uldall et al., 2017). However, these conflicting results can be attributed to several factors. Firstly, the role of CSF in contributing to elevated ICP may have limited relevance in patients with ABI, where reduced brain compliance because of cerebral edema is the predominant mechanism. Secondly, most previous studies were conducted in either healthy animals or patients with chronic neurological conditions, such as pseudotumor cerebri or chronic hydrocephalus, which are not directly comparable to acute cerebrovascular or traumatic brain injury. Thirdly, in this study, patients were consistently positioned with the head elevated at 30°, optimizing venous cerebral drainage to the heart. In contrast, the head position was not standardized in other studies (Ramos et al., 2024). Fourthly, our study focused on assessing the early effects of acetazolamide within a timeframe of 5–20 min, whereas previous studies evaluated ICP values at 1–4 h post‐treatment. Therefore, we are unable to draw conclusions regarding the long‐term effects of acetazolamide on ICP and PbtO_2_ in ABI patients. Lastly, it is worth noting that our patients were all under controlled mechanical ventilation, which might have influenced the final results. Patients who are spontaneously breathing have the ability to adjust their respiratory rate in response to a reduction in blood pH, mediated by the activation of the respiratory center in the brainstem; this respiratory compensation mechanism could potentially mitigate the vasodilatory effects of acetazolamide.

The second observed effect following cerebral vasodilation was a significant increase in PbtO_2_, effectively correcting tissue hypoxia in all patients except one. In particular, the effects of decreased perivascular pH on cerebral arterioles result in: (a) increased CBF; (b) increased arterial cerebral blood volume; (c) increased oxygen delivery to the tissue (Gouvea Bogossian et al., 2021, 2023; Westermaier et al., 2016). Also, the oxyhemoglobin dissociation curve was shifted to the right, thus displacing oxygen from hemoglobin to the tissues. The effects of acetazolamide on p50 are controversial. In 12 critically ill patients with metabolic alkalosis, intravenous acetazolamide (15 mg/kg body weight) did not significantly affect the p50 (Berthelsen, 1982). In another study, acetazolamide given over 2 days was associated with a significant increase in p50 when administered in healthy volunteers subsequently exposed to normobaric hypoxic chamber for 6 h (Liu et al., 2024). However, variations in total dosage, route of administration, patient conditions, and assessment methods of p50 were heterogeneous across studies, which could account for these findings. Our data highlight the potential therapeutic value of acetazolamide in improving PbtO_2_ in this setting; however, whether acetazolamide could be an alternative to further increase PaCO_2_ in protocolized therapeutic protocols based on brain oxygenation remains to be further validated. While hypercapnia can have side effects, such as pulmonary hypertension, arrhythmias, and hypotension due to systemic vasodilation (Qian, 2018), it is important to note that acetazolamide can also present several side effects, such as hypovolemia and electrolyte disturbances (i.e., due to its diuretic effects), kidney stones, vomiting, hearing impairment, and profound acidemia (i.e., prolonged treatment) (Schmickl et al., 2020; Shukralla et al., 2022). Importantly, it should be noted that the effects of acetazolamide on PbtO_2_ were found to be heterogeneous among patients. This heterogeneity could be attributed to various factors, including differences in CMRO_2_ among individuals, which plays a significant role in tissue oxygenation independent of CBF increase. Additionally, variations in microvascular function could also contribute to these findings, as microvascular function directly affects oxygen diffusion to the tissue, irrespective of global CBF and arterial oxygen content (Roy & Secomb, 2021).

This study has several limitations that should be acknowledged. Firstly, the cohort size was small, and the results may be influenced by local practices, limiting the generalizability of our findings. Similarly, the correlation between FVm and PbtO_2_ may be influenced by “outliers,” which have a more significant impact when the number of observations is limited. Additionally, subgroup analyses (e.g., traumatic vs. non‐traumatic injury) should be considered as hypothesis‐generating due to the very small sample sizes in each cohort. However, considering the rarity of this therapeutic approach, it would have been difficult to obtain a larger patient cohort. Additionally, the consistency of results across patients lends support to the observed findings. Secondly, the absence of a standardized protocol for administering acetazolamide in these patients may have introduced some selection bias, that is, based on individual clinical judgment, which could vary among healthcare providers. For instance, all patients exhibited baseline tissue hypoxia (e.g., PbtO_2_ <20 mmHg), which may have prompted the use of acetazolamide to correct alkalosis. Therefore, we cannot draw conclusions regarding the effects of acetazolamide in patients with metabolic alkalosis and normal PbtO_2_ values. Thirdly, our study did not assess the effects of repeated acetazolamide administrations or explore different dosage regimens to determine if the observed effects are dose‐dependent. Also, as this was a physiological study, we focused on obtaining repeated measurements during the early phase after acetazolamide administration. Given that these critically ill patients often required additional interventions (e.g., fluid administration, ventilator adjustments, vasopressor modifications), it would have been challenging to maintain all other parameters stable over a prolonged period. Similarly, clinically relevant outcomes and a comparison group were beyond the study's scope and were not considered in this study. As a first report on the effects of acetazolamide on PbtO_2_ and ICP, the next study would be to compare this intervention with other therapies aimed at increasing PbtO_2_ in ABI patients with tissue hypoxia and alkalosis. Fourthly, tissue oxygenation was only evaluated in the monitored area, and it remains unknown whether there was a redistribution of blood flow within the brain, potentially leading to hypoperfusion in other regions. Fifth, using a headset or fixation device for TCCD would have ensured consistent probe positioning, minimized motion artifacts, and allowed measurements to be taken from the same vessel location and insonation angle over time, thereby improving data reliability. However, we applied specific techniques to ensure identification of the same MCA segment and to minimize the insonation angle; the significant decrease in PI over time, which is unaffected by insonation angle, suggested that our findings were consistent with cerebral vasodilation. Importantly, the PI derived from TCCD measurements, although commonly used as a surrogate marker for cerebrovascular resistance, has several limitations (Bill et al., 2020). These include the influence of systemic hemodynamics (e.g., pulse pressure, heart rate, arterial stiffness), elevated intracranial pressure (ICP), and reduced arterial compliance, or the lack of specificity in certain pathological conditions. These factors limit the reliability of PI in accurately reflecting changes in cerebrovascular resistance, particularly in patients with brain injury, and the need to combine other measurements (e.g., FVm or ICP) in this context. Finally, we did not evaluate potential side effects of acetazolamide or assess long‐term neurological outcomes in treated patients. These aspects should be considered in future studies to gain a more comprehensive understanding of the benefits and potential risks associated with acetazolamide treatment in this patient population.

## 5 CONCLUSIONS

In this study, the administration of intravenous acetazolamide resulted in a significant increase in brain oxygenation among patients with brain injury. This improvement in oxygenation was accompanied by an increase in ICP and cerebral blood flow velocities, indicating the induction of cerebral vasodilation. These findings suggest that acetazolamide may serve as a valuable therapeutic option for enhancing brain oxygenation in this specific clinical context.

## AUTHOR CONTRIBUTIONS

AH, CS, EGB, and FST conceived and designed the research; AH and FST collected the data; CS and FST analyzed data; AH, CS, IC, EGB, SS, MA, and FST interpreted results; AH and CS drafted the manuscript; AH, CS, IC, EGB, SS, MA, and FST edited and revised manuscript; all authors approved final version of manuscript.

## FUNDING INFORMATION

No funding information provided.

## CONFLICT OF INTEREST STATEMENT

No conflicts of interest, financial, or otherwise, are declared by the authors.

## ETHICS STATEMENT

The study protocol was approved by the local ethics committee (Comité d'Ethique de l'Hôpital Erasme – study ID: SRB2022383); a written delayed consent was obtained from next of kin, as the administration of acetazolamide was determined independently of the study, and the repeated and serial assessments of clinically relevant variables were part of a standardized data collection process for various interventions administered to patients undergoing invasive multimodal neuromonitoring in the ICU.

## Data Availability

Data will be made available upon reasonable request.
